# Tibialis anterior analysis from functional and architectural perspective during isometric foot dorsiflexion: a cross-sectional study of repeated measures

**DOI:** 10.1186/s13047-015-0132-3

**Published:** 2015-12-18

**Authors:** Maria Ruiz Muñoz, Manuel González-Sánchez, Antonio I. Cuesta-Vargas

**Affiliations:** Departamento de Enfermería y Podología, Instituto de Investigación Biomédica de Málaga (IBIMA), Universidad de Málaga, Málaga, Spain; Departamento Ciencias de la Salud. Instituto de Investigación Biomédica de Málaga (IBIMA), Universidad de Jaén, Málaga, Spain; Departamento de Fisioterapia, Facultd de Ciencias de la Salud, Instituto de Investigacion Biomedica de Malaga (IBIMA), Grupo Clinimetria F-14 Universidad de Malaga, Andalucia Tech, Malaga, Spain; School of Clinical Sciences at Queensland University, Brisbane, Australia

**Keywords:** Ankle, Electromyography, Ultrasonography, Multiple Regression Analysis, Anterior Tibial Muscle

## Abstract

**Background:**

The purpose of the present study is to establish the relationship and degree of contribution between torque and sonomiography variables (pennation angle – muscle thickness), and electromyography variables (EMG_AreaUnderCurve_ – EMG_MaximalPeak_) of the tibialis anterior muscle during (TA) maximal and relative isometric foot dorsiflexion (IFD). Secondary aim: To determine the measurement’s reliability.

**Methods:**

Cross-sectional study. 31 participants (15 men; 16 women) performed IFD at different intensities (100, 75, 50, and 25 %) of the maximal voluntary contraction (MVC) (three times for each intensity). Outcome variables: To determine the torque, pennation angle, muscle thickness, EMG_MaximalPeak_, and EMG_AreaUnderCurve_. Statistical analysis: In order to test the measurement’s reliability, Cronbach’s alpha and standard error of the measurement were determined. An inferential analysis was carried out using Pearson correlations(*r*). For each contraction intensity, a multiple regression analysis was performed, where the dependent variable was torque and the independent variables were EMG_AreaUnderCurve_, EMG_MaximalPeak_, muscle thickness and pennation angle.

**Results:**

All outcome variables show excellent reliability. The highest correlation value was 0.955 (thickness 100 % – thickness 25 %). *R*^*2*^ values ranged from 0.713 (100 % MVC) to 0.588 (25 % MVC).

**Conclusion:**

The outcome variables demonstrated excellent reliability in terms of measuring IFD at different intensities. The correlations between all outcome variables were moderate-to-strong. TA functional and architectural variables have a significant impact on the torque variance during IFD at different intensities.

## Background

Foot dorsiflexion (FD) plays a very important role in balance control and during the gait cycle [1, 2]. The tibialis anterior (TA) muscle is the main FD muscle, and an increase in this muscle’s strength is associated with a reduction in the risk of falling [3]. Previous studies have shown that the heel strike and swing phases are the two phases with increased TA muscle activity [1, 2]. It has been shown that walking slower with less efficiency may be associated with alterations of the TA muscle [3, 4].

The in-depth study of musculoskeletal structures allows for the acquisition of large amounts of information whose analysis favours the understanding of behaviour in different situations or with different stimuli [5, 6]. In the laboratory, a load cell is the instrument most commonly used for recording muscle torque [7]. In order to understand the factors that contribute to joint movements, it is common for researchers to analyse the changes of the parameters depending on strength [7, 8], both from an anatomical and a neuromotor perspective [5–8].

From an architectural point of view, muscle thickness and pennation angle have been used in various studies as indicators of the force generated during muscle contraction using sonomyography (SMG) [9–12]. Furthermore, surface electromyography (sEMG) is the most commonly instrument used for functional analysis of the muscle [13, 14], since it allows for analysing the motor unit’s potential activation, assessment of muscle activity or muscle strength estimation [15], as well as having some additional benefits (compared with intramuscular electromyography): it is non-invasive and has applicability in situ, and can be used to assess greater muscle surface [16]. Frequently, within the analysis of electromyographic signals, the sEMG signal amplitude [17] and the temporal spectrum are considered [8]. In this sense, the peak of muscle activation and area under the curve muscle activation allow for analysing the muscle behaviour in an instant or over a period of time, respectively [8, 17].

Research has been carried out regarding the TA muscle during isometric [12, 13] and isotonic [14] contractions at different intensities using sEMG and SMG. However, regarding analysing the behaviour of the TA muscle during an isometric contraction at different intensities, no studies using ultrasound, sEMG and torque synchronised in a large sample have been found.

The main aim of this study was to establish the relationship and degree of contribution between torque and SMG variables (pennation angle and muscle thickness), and sEMG variables (area under the curve and maximal peak) of the TA muscle when performing maximal and relative isometric FD (IFD). The second aim of this study was to perform a reliability analysis of the outcome variables at different IFD intensities. The hypothesis of our study was that architectural and electromyographic variables would explain the variance of the TA muscle during IFD at different intensities. For the second aim, the hypothesis was that EMG and SMG would show a highly reliable protocol in its use on the TA.

## Methods

Thirty-one healthy young adults (15 men and 16 women) participated in this cross-sectional study of repeated measures. Participants were recruited from a community health centre in Malaga (Spain) through ads placed in the centre. Data were collected in a human movement analysis laboratory between April 2014 and December 2014. The participants’ average (mean ± standard deviation) age, height, and body weight were 26.9 ± 5.7 years, 1.74 ± 0.12 m and 68.04 ± 12.54 kg, respectively. Exclusion criteria for the study included participants aged less than 18 or more than 40 years, neuro-musculoskeletal limitations that prevent completion of the protocol, cognitive impairment from any aetiology, surgical intervention in the lower limbs over the past 12 months (prior to the recruitment), BMI ≥ 35 kg/m^2^, severe metabolic, cardiovascular or respiratory problem and current pregnancy. Prior to the study, each participant provided written informed consent. The ethics committee of the Faculty of Health Sciences granted ethical approval for the present study. The study complied with the principles laid out in the Declaration of Helsinki. The participants’ personal data were treated in accordance with the Spanish Organic Law of Protection for Personal Data 15/1999.

### Participants’ position

**Fig. 1:**
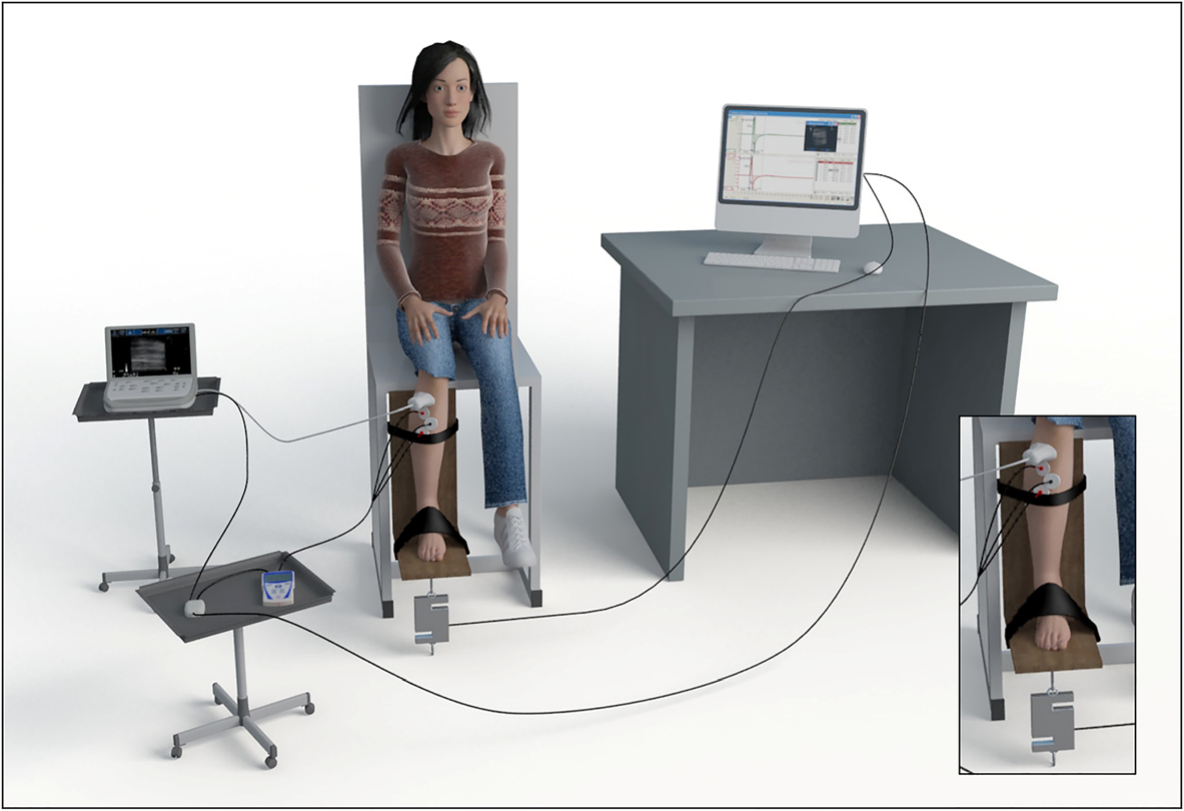
Scheme of the isometric position test and devices used. Scheme includes all devices and accessories: US and probe, trigger, electromyograph and electrodes, load cell and screen with EMG graphic registration

All participants sat with knees and hips flexed at 90°. From this position, each participant positioned the dominant foot on a device comprised of two platforms (one horizontal and one vertical) that allowed the foot to be placed in a neutral position (90° angle between the foot and leg) (Fig. 1). Velcro® straps were used to prevent leg and foot movement during the execution of IFD.

### Torque measurement

A load cell (Biomotor Mega 6000 accessories – Mega Electronics Ltd, Kuopio, Finland) [18] was positioned, connected by chains, between the horizontal platform and the ground (Fig. 1). To ensure the IFD, the load cell blocked the movement during FD. The torque measurement was performed throughout the duration of the IFD (five seconds). The maximum value measured during IFD was considered the maximum torque and was used to normalise the variables and analyse offline SMG and EMG variables.

### Ultrasound measurement

The Esaote MyLab25 Gold ultrasound system was used to acquire ultrasound. A probe model LA523 [19], with a frequency of 12 Hz at 5 cm deep, was used to acquire ultrasound images. An operator with extensive experience in musculoskeletal SMG performed the ultrasound image acquisition. The probe was placed on the right leg on the first third of the leg, parallel to the major axis of the TA muscle, using the tibial tuberosity as a reference (the probe was placed below) [13]. Before the study, extensive pilot testing with reference to anatomy textbooks, models and SMG studies [1,203] was conducted to determine the best scanning protocol.

### Electromyography measurement

Activation of the TA muscle was measured using the Biomonitor ME6000 electromyograph (Mega Electronics Ltd, Kuopio, Finland) [18] with a sampling frequency of 1000 Hz. The electrodes were positioned and the skin prepared in accordance with the European Recommendations for Surface Electromyography (sEMG) [2, 20, 21]. In order to avoid EMG crosstalk, special attention was paid to the placement of the electrodes, as well as to the spacing and size [2, 20]. A space was provided for placing the ultrasound probe without affecting the position and operation of the electrodes. The line between the medial malleolus and the fibula’s head was used to position the electrodes. After a TA muscle belly palpation, the electrodes were positioned in the proximal third (the reference line), with a distance between the electrodes of 2 cm. Following the electromyograph manufacturer’s protocol (Mega Electronics Ltd, Kuopio, Finland) [18]) for each participant, three Al/AgCl electrodes (5 cm in diameter) were used (two electrodes were used as poles, while the third electrode was used as a reference). To remove high-frequency noise, the signal was filtered via a low pass Butterworth filter (bidirectional fourth-order).

### Synchronisation data acquisition

The Megawin 3.0.1 software and a Biomonitor ME6000 console [Mega Electronics Ltd] [18] connected to each device were used to continuously and synchronously record the SMG and sEMG data. Torque was the reference variable used in order to subsequently measure SMG variables (thickness and pennation angle) and EMG variables of the TA. The start and end of the synchronising of all systems during each test were controlled using a trigger device [Mega Electronics] [18].

### Experimental procedure

For all participants, the measurements were performed on the dominant foot. Before the experimental procedure, each participant was able to perform all repetitions deemed necessary to become familiar with the protocol. Three maximal IFD (defined as the highest torque value measured [11]) were carried out for five seconds (The rest between each repetition was 90 s to prevent that fatigue that could influence contractions). Torque, SMG and EMG signals were collected during the test.

#### Relative contractions

The torque value recorded during maximal voluntary contraction (MVC) was used as a reference for calculating the intensity of the MVC relative contractions. Participants performed 75, 50 and 25 % of their torque recorded during MVC. Each participant performed three repetitions for each IFD relative contraction, with 90 s of rest between each repetition. All participants performed the same sequence of IFD (100 % – 75 % – 50 % – 25 % MVC). Torque, SMG (pennation angle and muscle thickness) and EMG variables (EMG_AreaUnderCurve_ (EMG_AUC_) and EMG_MaximumPeak_ (EMG_MP_)) were measured offline from the data collected during the protocol.

### Data analysis

The instant at which maximum torque was recorded was used as a reference for extracting the EMG and SMG variables. Architectural variables (thickness and angle pennation muscle) were calculated offline. The ultrasound pictures were imported into a specific programme for the processing and analysis of images (AutoCAD 2012 – English SP2 software (Autodesk, San Rafael, California, USA)). Pennation angle was considered as the distance between the central intramuscular septum and the line of the clearest fascicle was considered as the positive angle [9]. In addition, muscle thickness was considered as the distance between the superficial and deep muscle aponeuroses [9].

**Fig. 2:**
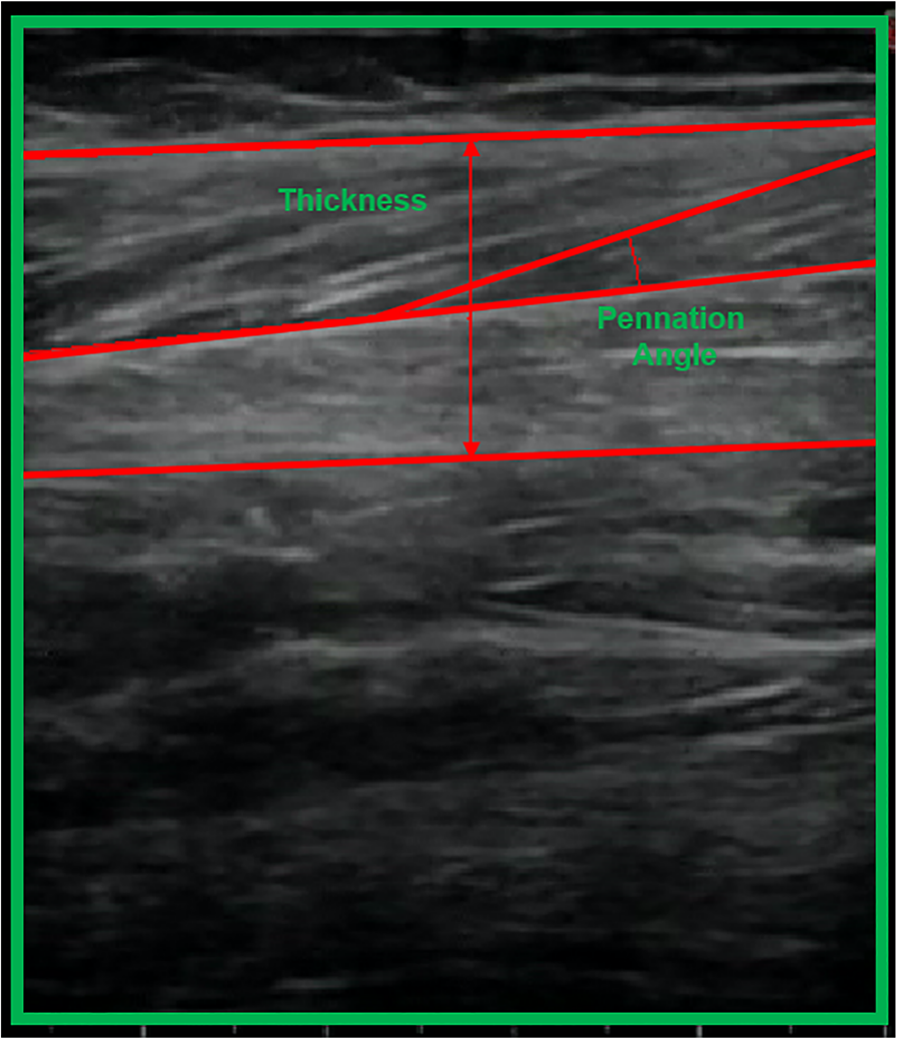
Example of pennation angle and muscle thickness measurement. Muscle thickness and pennation angle measurements of the TA muscle performed in the longitudinal plane

The software MegaWin 3.0.1 (Mega Electronics Ltd, Kuopio, Finland) [18] was used to record and process the EMG signal. The muscle functional variables (EMG_MP_ and EMG_AUC_) were extracted from the raw results of a selected area (encompasses the EMG_MP_ and one second before and after this time point). An independent and blinded researcher analysed the data (Fig. 2).

### Statistical analysis

The reliability (considered as a test–retest standard deviation of differences as the 95 % limits of agreement) was calculated for each outcome variable (torque, EMG_AUC_, EMG_MP_, muscle thickness and pennation angle). For this purpose, the three measures were acquired during each IFD intensity and were used to calculate the standard error of the measurement (SEM) and the internal consistency (Cronbach’s alpha) of the measure, together with the 95 % confidence interval for each variable. Reliability was classified as follows: excellent (Cronbach’s alpha: > 0.80), good (Cronbach’s alpha: 0.80–0.60), moderate (Cronbach’s alpha 0.60–0 .40), or poor (Cronbach’s alpha <0.40) [22, 23].

A descriptive statistical analysis was carried out using mean and standard deviation. An inferential analysis was carried out using a Pearson correlations(*r*) in accordance with the normality of the variables after a sample K–S test. For each contraction intensity, an exponential regression analysis was performed, where the dependent variable was torque and the independent variables were EMG_AUC_, EMG_MP_, muscle thickness and pennation angle. The magnitude of the correlations was as follows: strong (r > 0.75), moderate (0.50–0.74), or poor (r < 0.49) [24].

The software G Power (Version 3.1) was used to estimate the sample size. In the a priori calculation, based on the literature [13], a minimum of 26 subjects was necessary to have sufficient statistical power (80 %) and an alpha error of 0.05. The statistical analysis was carried out using the SPSS 21.0 statistical package for Windows.

## Results

All outcome variables show an excellent reliability level, with Cronbach’s alpha values ranging from 0.998 (torque) to 0.901 (EMG_AUC_) at 100 % MVC, from 0.996 (torque) to 0.878 (EMG_MP_) at 75 % MVC, 0.991 (torque) to 0.869 (EMG_AUC_) at 50 % MVC and 0.980 (torque) to 0.980 (EMG_MP_) at 25 % MVC, respectively. The remainder of the Cronbach’s alpha values and SEMs are shown in Table 1.

**Table 1:** Reliability of torque, SMG variables (thickness and pennation angle) and EMG (EMG_AUC_, EMG_MP_) variables of the TA during each isometric foot dorsiflexion intensity

Isometric foot dorsiflexion intensity		100 %	75 %	50 %	25 %
Thickness	Cronbach’s α (IC 95 %)	0.994 (0.989–0.998)	0.987 (0.980–0.992)	0.986 (0.978–0.990)	0.981 (0.969–0.987)
	SEM (Stand. Error. Measu.)	0.687	0.784	0.822	0.867
Penattion Angle	Cronbach’s α (IC 95 %)	0.992 (0.986–0.998)	0.984 (0.976–0.989)	0.982 (0.974–0.987)	0.973 (0.955–0.984)
	SEM (Stand. Error. Measu.)	0.389	0.404	0.428	0.477
EMG_MP_	Cronbach’s α (IC 95 %)	0.917 (0.880–0.932)	0.878 (0.970–0.991)	0.877 (0.859–0.893)	0.851 (0.920–0.968)
	SEM (Stand. Error. Measu.)	5.193	5.479	5.204	5.388
EMG_AUC_	Cronbach’s α (IC 95 %)	0.901 (0.872–0.921)	0.881 (0.871–0.908)	0.869 (0.848–0.883)	0.857 (0.829–0.866)
	SEM (Stand. Error. Measu.)	7.587	7.690	7.555	7.316
Torque	Cronbach’s α (IC 95 %)	0.998 (0.994–0.999)	0.996 (0.980–0.998)	0.991 (0.982–0.997)	0.980 (0.967–0.995)
	SEM (Stand. Error. Measu.)	0.503	0.376	0.353	0.112

*EMG* electromyography, *TH* thickness, *PA* pennation angle, *MP* maximum peak, *AUC* area under the curve

The results presented in Table 2 include the average of the registrations performed during the three repetitions of the protocol. Records of all outcome variables (torque, EMG_AUC_, EMG_MP_ muscle thickness and pennation angle) increased progressively with increasing contraction intensity (from 25 % to 100 % MVC), and mean values ranging from 17.29–21.33 mm (muscle thickness), 4.90–11.83° (pennation angle), 190.26–546.35 μV (EMG_MP_), 241.78–721.48 μV (EMG_AUC_) and 8.34–49.87 Nm (Torque) (Table 2).

**Table 2:** Descriptive data of the tibialis anterior muscle during maximal and relative isometric foot dorsiflexion

Isometric	100 %	75 %	50 %	25 %
	Mean (Minimum-Maximum)			
Thickness (mm)	21.33 (12.37–35.36)	20.25 (11.7–28.56)	18.99 (11.13–24.87)	17.29 (9.39–20.5)
Penattion Angle (degrees)	11.83 (6–18)	10.10 (6–15)	7.60 (4–13)	4.90 (2–8)
EMG_MP_ (uV)	546.35 (216–918)	468.17 (161–747)	343.39 (138–630)	190.26 (2–469)
EMG_AUC_ (uV)	721.48 (241–1113)	624.96 (244–1085)	456.22 (86–905)	241.78 (4–470)
Torque DF Ankle (N · m)	49.87 (40.75–56.99)	34.49 (27.54–43.47)	24.23 (19.28–35.62)	8.34 (4.01–18.02)

*EMG* electromyography, *DF* dorsiflexion, *MP* maximum peak, *AUC* area under the curve

The present study shows that there was a moderate-to-strong correlation between the EMG activity, the architectural variables (muscle thickness and pennation angle) and the torque of the foot for the TA muscle. The highest correlation indices were among the same variables measured at different contraction intensities with 0.955 (thickness 100 % – thickness 25 %) being the highest correlation value. Correlations between all outcomes are shown in Table 3.

**Table 3:** Correlations between SMG variables, EMG variables and torque of TA for each isometric foot dorsiflexion intensity

	TH 100	TH 75	TH 50	TH 25	PA 100	PA 75	PA 50	PA 25	EMG 100	EMG 75	EMG 50	EMG 25	Area 100	Area 75	Area 50	Area 25	Torque 100	Torque 75	Torque 50	Torque 25
TH 100	1																			
TH 75	0.805†	1																		
TH 50	0.886†	0.886†	1																	
TH25	0.955†	0.882†	0.903†	1																
PA 100	0.687†	0.770†	0.743†	0.804†	1															
PA 75	0.560†	0.693†	0.593†	0.649†	0.712†	1														
PA 50	0.754†	0.779†	0.812†	0.816†	0.827†	0.839†	1													
PA 25	0.767†	0.744†	0.718†	0.849†	0.895†	0.750†	0.895†	1												
EMG_MP_ 100	0.650†	0.871†	0.776†	0.725†	0.634†	0.625†	0.765†	0.643†	1											
EMG_MP_ 75	0.131	0.299	0.204	0.164	0.296	0.402*	0.353	0.326	0.421*	1										
EMG_MP_ 50	0.678†	0.698†	0.624†	0.687†	0.515†	0.545†	0.588†	0.615†	0.748†	0.552†	1									
EMG_MP_ 25	0.556†	0.483*	0.439*	0.591†	0.584†	0.617†	0.577†	0.659†	0.446*	0.583†	0.693†	1								
EMG_AUC_ 100	0.586†	0.495†	0.548†	0.556†	0.426*	0.488†	0.590†	0.519†	0.593†	0.500†	0.767†	0.697†	1							
EMG_AUC_ 75	0.084	0.224	0.127	0.064	0.185	0.321	0.280	0.231	0.377	0.834†	0.505†	0.548†	0.579†	1						
EMG_AUC_ 50	0.544†	0.447*	0.379	0.525†	0.379	0.391*	0.379	0.464*	0.448*	0.416*	0.762†	0.628†	0.634†	0.410*	1					
EMG_AUC_ 25	0.500†	0.358	0.315	0.436*	0.398*	0.429*	0.441*	0.569†	0.326	0.571†	0.651†	0.760†	0.682†	0.686†	0.674†	1				
Torque 100	0.451*	0.500†	0.381	0.495†	0.398*	0.596†	0.587†	0.570†	0.468*	0.300	0.469*	0.608†	0.501†	0.392*	0.460*	0.544†	1			
Torque 75	0.188	0.282	0.175	0.217	0.268	0.441*	0.393*	0.388*	0.361	0.771†	0.481*	0.490†	0.424*	0.705†	0.407*	0.627†	0.474*	1		
Torque 50	0.426*	0.476*	0.353	0.407*	0.288	0.354	0.372	0.423*	0.467*	0.456*	0.689†	0.456*	0.532†	0.508†	0.638†	0.679†	0.701†	0.648†	1	
Torque 25	0.536†	0.490†	0.368	0.562†	0.479*	0.491†	0.483*	0.582†	0.408*	0.505†	0.657†	0.779†	0.589†	0.530†	0.618†	0.792†	0.555†	0.588†	0.596†	1

* Correlation is significant at the 0.05 level

† Correlation is significant at the 0.01 level

*EMG: electromyography. DF: dorsiflexion. TH: thickness. PA: pennation angle; MP Maximum peak; AUC: Area under the curve

A multiple regression analysis was performed using torque as the dependent variable and EMG and SMG variables as the independent variables; the *R*^*2*^ values were 0.713, 0.682, 0.627 and 0.588 for IFD at 100, 75, 50 and 25 % of MVC, respectively (Table 4). All variables contributed significantly to explaining the variance of the dependent variable at 100 % contraction. However, during lower intensity contractions (25–50 %), EMG variables made the most significant contribution to the variance of the dependent variable.

**Table 4:** Analysis of the degree of contribution of each independent variable to the dependent variable using a multiple regression

Dependent variable	Foot dorsiflexion Intensity (% MVC)	Predictor independent variables (standardised Beta)				R^2^	R^2^ Corrected
		EMG		SMG			
		Max. peak	AUC	PA	MT		
Torque	100 %	0.838†	0.807†	0.584*	0.607*	0.713	0.649
Β coef. No	75 %	0.670†	0.410*	0.219*	0.134	0.682	0.506
Stand.	50 %	0.515*	0.317*	−0.140	−0.014	0.627	0.576
4.376	25 %	0.786†	0.276*	0.025	−0.067	0.588	0.535

*MVC* maximal voluntary contraction, *EMG* electromyography, *SMG* sonomyography, *Max. Peak* maximum peak, *AUC* area under the curve, *PA* pennation angle, *MT* muscle thickness

Signification Level: * ≤ 0.05; † ≤ 0.01

## Discussion

The present study aimed to establish the relationship and degree of contribution between torque and SMG variables (thickness and pennation angle) and EMG (EMG_AUC_, EMG_MP_) variables of the TA during maximal and relative IFD. In addition, the present study aimed to analyse the reliability of the outcome variables at different IFD intensities. According to our results, the degree of contribution between torque and EMG and SMG variables increases with the intensity of the IFD, whereas the relationship between the outcome variables is moderate. In addition, the reliability level was good for all outcome variables along all intensities of IFD. It is also possible to affirm that the objectives of the present study were achieved, further confirming the hypothesis.

The current research shows that pennation angle, muscle thickness, EMG_MP_ and EMG_AUC_ explain 71.3 % of the variance in the torque during maximal IFD and 68.2, 62.7 and 58.8 % for 75, 50 and 25 % of maximal IFD, respectively. A possible explanation for the variance in torque during IFD that is not explained by the variables considered in this study could be found in the participation of the other foot’s dorsal flexors (extensor of the ankle muscle and extensor of the hallux muscle), which also determine the torque recorded during IFD. In addition, muscle fibre type, subcutaneous fat, and proper limits of the sEMG methodology may be related to the remaining unexplained variance in this model [2, 25]. These drawbacks include interference or cross-talk from adjacent muscles [2, 13, 25], as well as the positioning of electrodes.

To our knowledge, this is the first study to analyse participation of the pennation angle, muscle thickness, EMG_MP_ and EMG_AUC_ of the TA muscle to explain torque variance during IFDs at different intensities. However, other studies have analysed the TA muscle and assessed the relationship between some variables that were considered in this study [12–14]. Manal et al. [12] used these analyses to predict pennation angle from sEMG. Hodges et al. [13] used the same analysis and also included muscle thickness. Ruiz-Muñoz and Cuesta-Vargas [14] analysed the degree of contribution of the TA pennation angle and muscle thickness on the variance of EMG_PeakActivation_ during isotonic FDs. Hodges et al. [13] and Manal et al. [12] found a strong relationship (*R*^2^ = 0.96 and *R*^2^ = 0.76, respectively) between EMG and the pennation angle of the TA muscle. A strong relationship (*R*^2^ = 0.75) was also found between EMG and muscle thickness [12]. In addition, Ruiz-Muñoz and Cuesta-Vargas [14] found a strong relationship between TA EMG_PeakActivation_, TA muscle thickness and pennation angle (*R*^2^ = 0.693) during isotonic FD. In the present study, the *R*^*2*^ value exceeded 70 % for the maximum IFD (71.3 % (MVC)), while for the other contraction intensities, the *R*^*2*^ values fell slightly (Table 4).

These studies [12, 13] synchronised EMG, SMG and torque in groups of healthy subjects, including one group consisting of five participants (four men and one woman) and one group with 16 participants (eight men and eight women), respectively. In the present study, the number of participants was increased to 31 (15 men and 16 women). Moreover, Maganaris and Baltzopoulos [26] analysed changes in the TA muscle pennation angle when going from rest to maximal isometric contraction, finding variations above 60 % [26]. However, this study, where the initial position and final positions were -15° of foot dorsiflexion and +30° of plantar flexion, respectively, there were no significant changes in TA thickness during MVC [26].

The present study shows that muscle thickness and the pennation angle of the TA muscle demonstrate a moderate-to-strong correlation with the EMG muscle. Moreover, there is also a moderate-to-strong correlation between architectural variables and torque, which means that muscle thickness and pennation angle increase proportionally as torque increases. Therefore, if in a specific injured muscle, these SMG parameters increase after an intervention, the strength of that muscle will also increase so that the intervention will be effective. Between EMG and the pennation angle, similar significant relationships were found in other muscles of the lower limbs (i.e. the three heads of the triceps sural (soleus, lateral and medial gastrocnemius: *r* > 0.80) [12]. Some previous studies, which have linked muscle architecture and electrical activity in the muscles of the upper limbs [3, 26], found moderate-to-strong correlations between sonographic and electromyographic variables. Furthermore, others researchers studied the trunk [13, 27], where a correlation was found between sEMG and SMG in the transverse abdominal muscles (*R* = 0.90) and internal oblique muscles (*R* = 0.84), but not in the external oblique muscles; however, others [27] found no relationship in these muscles (*r* ≤ 0.14). Nevertheless, the strong correlation between EMG and SMG variables observed in the present study of the TA, when participating in an IFD at different intensities, is not consistent when the same muscle participates in an isotonic FD, where the correlation between EMG and SMG variables was not significant [14].

The reliability level of muscle thickness observed in this study (0.994 (95 % CI 0.989–0.998)) is consistent with that obtained in McCreesh et al. [28] and Cartwright et al.’s [29] studies, with values of 0.994 (95 % CI 0.985–0.998) and 0.984 (CI not available), respectively. Bland et al.’s [9] study of subjects with cerebral palsy analysed, in a brief manner, the reliability levels of the architectural variables investigated in the current study. The author stated that reliability levels were high, ranging between 0.98 and 0.99, but the author did not specify which value corresponded to each of the architectural variables. Regarding the TA muscle, a study aimed at analysing the reliability of SMG’s use for obtaining muscle architecture variables specifically and comprehensively during IFD was not found. However, a previous study analysed the reliability of this variable during isotonic FD [14], with values of ICC = 0.991 (0.979–0.996), for muscle thickness, and ICC = 0.910 (0.795–0.960) for pennation angle. These reliability levels are consistent with the range of the reliability observed in the architecture variables of the TA during IFD, where levels varied between 0.994 and 0.981 for muscle thickness and 0.992 and 0.973 for pennation angle (Table 1).

### Study Limitations

The present study has some limitations that must be taken into account when interpreting the results. The MVC can be highly dependent on the level of motivation of the subject, and therefore, the recorded values cannot be considered as absolute values, since the subjects, despite being healthy, could present different levels of motivation. This could condition the MVC record and its relative activation levels (75, 50, and 25 % MVC). In addition, there are limits with the use of sEMG, such as altering the electromyographic signal due to subcutaneous fat, cross-talk or skin impedance. While the authors tried to minimise these limits following the recommendations of SENIAM [21] (excluding participants with a BMI of 35 kg/m^2^, shaving the skin and removing dead skin cells and preparing the skin with alcohol), it is important to consider these limits and not take the results as absolute measures. In addition, future studies could increase the number of participants to improve consistency in the results. In addition, they could also analyse the behaviour of the non-dominant leg in order to compare the performance with the dominant leg.

## Conclusions

In the proposed model, EMG_AUC_, EMG_MP_, muscle thickness and pennation angle explained 71.3 % of the torque variance reached by the TA muscle during maximal IFD. Their participation in the variance of the dependent variable is slightly smaller in lower contraction intensities. The correlations between torque, EMG and architectural variables were moderate-to-strong. In addition, torque, SMG variables (thickness and pennation angle) and EMG (EMG_AUC_, EMG_MP_) demonstrated excellent reliability for measuring IFD at different intensities.

## Abbreviations

EMG: Electromyography
EMG_AUC_: EMG_AreaUnderCurve_
EMG_MP_: EMG_MaximumPeak_
FD: Foot Dorsiflexion
IFD: Isometric Foot Dorsiflexion
MVC: Maximal Voluntary Contraction
SEM: Standard Error of the Measurement
sEMG: Surface Electromyography
SMG: Sonomiography
